# Improved treatment outcomes with adalimumab therapeutic monitoring in granulomatous cheilitis

**DOI:** 10.1016/j.jdcr.2026.05.067

**Published:** 2026-06-05

**Authors:** Faith Simmonds, Alexandra Coromilas

**Affiliations:** a Columbia University Vagelos College of Physicians and Surgeons, New York, New York; b Department of Dermatology, Columbia University Vagelos College of Physicians and Surgeons, New York, New York; c NewYork-Presbyterian Columbia University Irving Medical Center, New York, New York

**Keywords:** adalimumab, drug response, granulomatous cheilitis, medical dermatology, therapeutic drug monitoring

## Introduction

Granulomatous cheilitis (GC) is a rare subtype of orofacial granulomatosis characterized by painless, recurrent edema of the lips. Histologically, it is defined by the presence of noncaseating granulomatous infiltrates with associated lymphedema and fibrosis.^1^ The etiology of GC remains poorly understood; however, previous studies have suggested potential genetic, immune-mediated, allergic, and microbial mechanisms.^1^ To date, there is no established standard of care treatment for GC. Several therapeutic agents have been trialed with variable efficacy, including intralesional and systemic corticosteroids, clofazimine, hydroxychloroquine, antibiotics, and, more recently, biologics.^2^

Adalimumab, a monoclonal antibody targeting TNF-α, has been increasingly used for GC refractory to other treatments. Despite its efficacy, higher doses may be needed to achieve therapeutic response, and decreasing therapeutic response over time has been reported with TNF-α inhibitors across multiple disease indications.^3^, ^4^, ^5^

Therapeutic drug monitoring (TDM) is considered a promising strategy to optimize treatment outcomes. However, there has been limited use of TDM in the field of dermatology, particularly for GC. Herein, we present a case of a patient with GC treated with adalimumab who developed increased disease activity that was successfully managed through TDM-guided dose adjustment. This case highlights the potential utility of TDM in optimizing biologics for inflammatory dermatologic conditions especially for those in which treatment options remain limited.

## Case report

A 60-year-old healthy woman initially presented to a rheumatology clinic with acute-onset swelling of the lower lip with an associated erythematous rash of the perioral skin for 16 months. She denied any gastrointestinal or respiratory symptoms. She was referred to dermatology and diagnosed with GC based on clinical presentation as well as non-caseating granulomatous inflammation on lip biopsy. Following her diagnosis, a chest x-ray, colonoscopy, and endoscopy were performed to exclude alternative diagnoses, such as subclinical inflammatory bowel disease and sarcoidosis, all of which were unremarkable. She was treated with various topical steroid ointments, tacrolimus ointment, as well as doxycycline and metronidazole with no substantial impact on her symptoms.

Initial treatment included 2 courses of prednisone, but she continued to experience intermittent flares. Adalimumab therapy was initiated as a steroid sparing agent, following inflammatory bowel disease dosing guidelines at 40 mg every other week. In addition, she was treated with topical tacrolimus, an additional prednisone taper, and doxycycline. Two months after initiation of adalimumab, she presented to our clinic, where she was found to have ongoing disease activity with diffuse lower lip edema without intraoral lesions. Laboratory testing showed an adalimumab level of 8.6 *μ*g/mL with no detectable anti-adalimumab antibodies. Her regimen was continued as she noted improvement from prior and preferred to allow more time for adalimumab to reach full therapeutic efficacy.

Despite initial improvement, 6 months after initiating adalimumab, she developed acute, worsening swelling and erythema of her lower lip. She had no new exposures or dietary changes. Repeat adalimumab level measurements revealed a trough of 6 *μ*g/mL and low-level anti-adalimumab antibodies at 3.6 *μ*g/mL. In accordance with American Gastroenterological Association recommendations for inflammatory bowel disease,^6^ her adalimumab dose was increased to 40 mg weekly to reach a target trough greater than or equal to 7.5 *μ*g/mL. The patient preferred not to initiate methotrexate for treatment of detectable anti-adalimumab antibodies.

**Fig 1:**
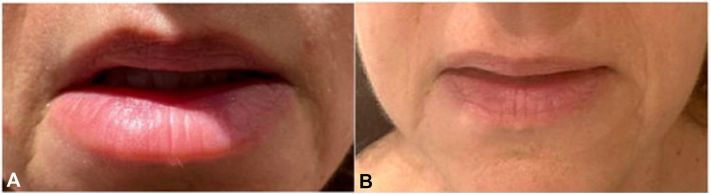
Granulomatous cheilitis. **A,** Acute granulomatous cheilitis flare before TDM-guided dose adjustment. **B,** Granulomatous cheilitis after TDM-guided dose adjustment.

Three months after the dose increase, the adalimumab trough increased to 11.6 *μ*g/mL with no detectable anti-adalimumab antibodies. She has since experienced sustained clinical improvement with resolution of swelling and no disease flares for 13 month following this therapeutic adjustment (Fig 1).

## Discussion

We present a case of GC with suboptimal response to adalimumab that was successfully optimized through TDM-guided dose adjustment. GC is thought to develop through heightened Th1-mediated inflammatory responses, leading to increased TNF-*α* production and subsequent mucosal damage.^2^^,^^7^ Thus, adalimumab is thought to be particularly efficacious in the treatment of GC. However, biologic immunogenicity remains a major limitation to its continued use. This is largely explained by the production of anti-drug antibodies, which promotes increased drug clearance and reduced target binding.^8^ Loss of adalimumab response has been well-documented across several inflammatory diseases, including psoriasis, inflammatory bowel disease, and rheumatic arthritis, where subsets of patients were found to have detectable anti-adalimumab antibodies.^3^, ^4^, ^5^

TDM offers a promising approach to mitigate loss of adalimumab efficacy. TDM can be implemented proactively, where serum drug concentrations are measured regardless of clinical response, or reactively, in which levels are only measured in patients showing partial or no response.^8^ In a retrospective study of adalimumab-treated psoriasis, TDM resulted in dose escalation and restored responsiveness in participants with subtherapeutic levels or detectable anti-adalimumab antibodies; additionally, proactive TDM resulted in dose reduction in responsive patients with supratherapeutic adalimumab levels.^3^

Recognizing its clinical utility in the field of dermatology, a proposed algorithm for TNF-inhibitor TDM in dermatologic disease management suggests that assessing therapeutic levels in patients with suboptimal responses can then guide the need for additional anti-drug antibody assays, dose escalation, or an alternative biologic class.^8^ To our knowledge, this is the first published report of TDM in adalimumab-treated GC. Although this patient has maintained a therapeutic response, long-term monitoring will be required to ensure sustained treatment efficacy and to assess for the redevelopment of anti-drug antibodies.

Of note, perioral erythema has been described in approximately 30% of cases in a cohort of orofacial granulomatosis.^9^ Given that GC is the most common presentation of orofacial granulomatosis, perioral erythema, as observed in this case, may also occur in GC despite its more typical association with nonerythematous lip swelling.

In conclusion, this case highlights the potential of standardized TDM to enhance treatment responsiveness and improve outcomes in chronic inflammatory conditions such as GC, which otherwise have limited therapeutic options.

## Conflicts of interest

None disclosed.
